# Editorial: Risk and Protective Factors Associated With Early Adversity and Development: Evidence From Human and Animal Research

**DOI:** 10.3389/fpsyg.2019.02906

**Published:** 2020-01-08

**Authors:** Livio Provenzi, Rosario Montirosso, Ed Tronick

**Affiliations:** ^1^0-3 Center for the at-Risk Infant, Scientific Institute IRCCS E. Medea, Lecco, Italy; ^2^Developmental and Brain Sciences Program, Department of Psychology, University of Massachusetts Boston, Boston, MA, United States; ^3^Department of Newborn Medicine, Harvard Medical School, Boston, MA, United States

**Keywords:** adverse life events, behavioral epigenetics, brain, development, parenting, preterm birth, social touch, stress

The recent advances at the crossroad among behavioral science, psychoneuroendocrinology, neuroscience, genetics, and epigenetics are revealing how early environmental exposures—for good or ill—leave biochemical “scars” or signatures in our developmental biology. These biological and behavioral memorial processes of our precocious encounters with life constitute the interwoven mechanisms that contribute to the long-lasting programming of health and disease later in life. The quality of early caregiving (Weaver et al., 2004; Conradt et al., 2016; Lester et al., 2018) and the exposure to adverse life events (McCrory et al., 2010; Blaze et al., 2015; Provenzi et al., 2016)—even when the exposure occurs in previous generations (Yehuda et al., 2005; Moog et al., 2016)—are embedded into our developing phenotype and contribute to our health trajectories and disease risk from infancy to adulthood.

The rapid and consistent growth of this field is seen in the birth of new translationally relevant scientific areas, e.g., behavioral epigenetics (Lester et al., 2011), microbiome research (Robertson et al., 2019), and in new ramifications of existing and well-established areas, e.g., psychoneuroendocrinology (Heim et al., 2019), social and affective neuroscience (Cascio et al., 2019), behavioral neuroimmunology (Suárez-Álvarez et al., 2013). Future advances in these areas of scientific investigation will greatly contribute to our comprehension of the pathways of disease risk and inform smarter and more effective preventive and therapeutic early interventions, finally enabling us to use the environmental-sensitive nature of our genes and biology to serve neuroprotective goals (Heim et al., 2019).

From this perspective, in the present Research Topic, we aimed at collecting evidence from both animal models and human studies on these emerging research efforts which are directed at disentangling how these mechanisms (e.g., epigenetics, microbiome) may be “informed” by early environmental encounters and ultimately lead to specific health and disease outcomes. The 15 papers included in this issue vary widely in terms of subjects, exposures, mechanisms, and outcomes. The collection includes original research articles, theoretical viewpoints, systematic reviews, and methodological issues. Here, our hope was to make order out of this complexity by assuming a developmental view inspired by a dynamic system epistemological approach to early development (van Geert, 2008). The dynamic system approach is a theory of embodied and embedded actions that considers developmental processes as emerging from the continuous coupling between the organism and the environment. More specifically, the early, continuous and fine-tuned micro-regulatory processes occurring between the individual and the environment result in the establishment of stable, yet flexible attractor states that further contributes to define the developmental trajectories later in life (Smith and Thelen, 2003). In other words, development is considered as the byproduct of messy and reciprocal connections (and disconnections) between an individual and the physical and conspecific environment in which he/she is situated (Tronick, 2017). Environmental encounters—such as the quality of parental caregiving or the exposure to traumatic events—play a critical role as they contribute to the shaping of the complex weaving of connections that result in the emerging behavioral phenotype of a given individual. Notably, from this viewpoint, environmental encounters cannot be defined as positive or negative *per sè*. Rather, encounters may provide beneficial or detrimental contributions for the developmental trajectory of a living being depending on the biochemical, cognitive, and socio-emotional that embed their effects in the developing biology and behavior of that given individual. Most prominently, reparation processes at many levels of the individual—molecular to behavioral—of messy or disruptive encounters with the human and physical environment assume a critical role by increasing the complexity and coherence of these processes which ultimately contributes to better and healthier outcomes later in life (DiCorcia and Tronick, 2011).

The original articles published in this Research Topic highlight the potential of early adversities occurring from pregnancy (Zietlow et al.) to the perinatal period (Gonzalez-Valenzuela et al.; Muntsant et al.) and from childhood (Stack et al.; Vilaseca et al.) up to adulthood (Liu et al.; Ranger et al.). Moreover, evidence of the impact of positive caregiving on infants' brain development (Hanford et al.) and cognitive and behavioral outcomes (Lejeune et al.) has is also reported. Taken together, these findings highlight the messiness of the dynamic developmental framework in which a living being grows in complexity as well as the openness of the developing phenotype to a wide and diverse set of potential environmental encounters. These encounters range from perinatal brain damage (Muntsant et al.) to postnatal music exposure (Lejeune et al.), from parental sensitive caregiving (Stack et al.; Vilaseca et al.; Zietlow et al.) to painful stimulations (Ranger et al.). The exposure to such a variety of environmental exposures is capable of shaping of cognitive development (Lejeune et al.; Muntsant et al.), brain connectivity (Hanford et al.), emotional (Stack et al.), and neuroendocrine regulation (Zietlow et al.) and even social and mating behavior in adult life (Liu et al.).

The theoretical, review, and methodological papers collected in this Research Topic further contribute by putting emerging research in the field into a broader context and by providing a different perspective for interpreting the research findings and highlighting clinical implications. Ludwig and Welch discuss controversies in the Darwinian theoretical construction. They ambitiously propose two testable theoretical arguments that move from lessons learned in clinical practice with at-risk babies which inform a relational and epigenetic model of emotional development that can be beneficial for early assessment and interventions in infancy and childhood. Two reviews focus on the embedding of early life stress into the developmental trajectories of behavioral, neuroendocrine, and immune system regulation. The mini review from Fogelman and Canli underlines the neuroendocrine and immune system physiological processes which contribute to the long-term embedding of early environmental encounters. Ylijoki et al. systematically reviewed evidence of the prenatal risk factors involved in setting the risk for detrimental health outcomes in preterm infants. Filippa et al. further focus on a specific potential mechanism—oxytocin secretion and its regulation—that may help us in understanding how early parental engagement may be beneficial for healthy development of preterm infants exposed to repeated painful stimulation during the hospitalization in the neonatal intensive care unit.  Sacchi et al. present a brilliant study protocol that holds potentials for revealing how the post-natal caring environment may be beneficial for the socio-cognitive development in infants with intrauterine growth restriction, at least partially through neural mechanisms involved in social and non-social stimuli elaboration. Finally, a review of methodological approaches used in the field of developmental human behavioral epigenetics is provided by Provenzi et al., who highlight the pros and cons of different retrospective and prospective research architectures for the advancement of the field.

In conclusion, the present Research Topic successfully collected contributions from researchers and clinicians working in different European (i.e., Germany, Finland, Italy, Spain, Switzerland), Asiatic (i.e., China), and North American (i.e., Canada, United States of America) countries. The virtuous integration of expertise between the scientific and the clinical frameworks is hugely needed in order to advance the scientific knowledge in directions that can forcefully impact our capacity to provide smarter interventions and more efficient preventive strategies for at-risk individuals. We hope that the present Research Topic may be a step forward into this direction and that it can benefit future translational studies.

## Author Contributions

All authors listed have made a substantial, direct and intellectual contribution to the work, and approved it for publication.

### Conflict of Interest

The authors declare that the research was conducted in the absence of any commercial or financial relationships that could be construed as a potential conflict of interest.
